# Detection and Isolation of Tissue‐Specific Extracellular Vesicles From the Blood

**DOI:** 10.1002/jex2.70059

**Published:** 2025-06-22

**Authors:** Lauren Newman, Andrew Rowland

**Affiliations:** ^1^ College of Medicine and Public Health Flinders University Bedford Park South Australia Australia

**Keywords:** cell origin, EV subtypes, extracellular vesicles, immunoaffinity isolation, organ‐derived, tissue‐specific

## Abstract

Extracellular vesicles (EVs) are nanosized, membrane‐bound particles released by virtually all cell types, serving as messengers within tissues and across organs via the bloodstream. EVs encapsulate diverse molecular cargo that reflects the phenotypic state of their originating cells, making them promising candidates for liquid biopsy applications. However, the heterogeneity of circulating EVs, comprising particles from various cell types and non‐vesicular entities like lipoproteins, poses significant challenges for isolating tissue‐specific EV populations. This review examines current methodologies for detecting and isolating tissue‐specific EVs from blood, focusing on immunoaffinity capture (IAC) strategies that leverage surface marker expression for specificity. Key considerations, including the selection and validation of markers, are discussed alongside advances in EV subtyping and isolation protocols. Challenges such as marker cross‐reactivity, EV biogenesis and transport dynamics are highlighted to underscore the complexity of achieving clinical utility. By providing an overview of validated tissue‐specific markers and isolation techniques, this review aims to facilitate the development of EV‐based biomarkers with enhanced specificity and sensitivity, enabling minimally invasive monitoring of organ function and disease.

## 1 Introduction

Virtually all cell types in the body release populations of nanosized membrane‐bound particles, known as extracellular vesicles (EVs), that encapsulate a diverse array of molecular cargo derived from their parent cells. EVs released into the interstitial space act locally within tissues as cellular messengers, with critical roles in maintaining homeostasis (Watanabe et al. 2022). As they may also travel to distant tissues, EVs participate in a complex network of interorgan communication, circulating via the superhighways of the vasculature (Iannotta et al. 2024). During biogenesis and secretion, EVs inherit nucleic acids, proteins, lipids, metabolites and other cargoes, creating a temporal snapshot of the releasing cell's molecular and phenotypic status (Dixson et al. 2023). Within the bloodstream, the lipid bilayer structure protects its cargo, preserving a wealth of biomarkers with potential relevance for understanding physiology and disease mechanisms. Thus, the presence of EVs in the blood derived from different tissues presents the opportunity to gather molecular information from organs in a minimally invasive liquid biopsy. This is a particularly attractive prospect for hard‐to‐access tissues, such as the brain, and to facilitate longitudinal monitoring (Mustapic et al. 2017).

However, once EVs enter the circulation, they join a heterogenous mixture of extracellular particles including EVs from other cell types and non‐vesicular entities such as lipoproteins and soluble proteins. This heterogeneity complicates the attribution of EVs to specific tissue origins, as most methods to prepare EVs from biofluids capture the bulk of particles with low specificity, and represents a critical hurdle for establishing the relevance to disease and clinical translation of EV‐based diagnostics (Shah et al. 2018). Although markers have been proposed for tissue‐specific EV populations, the rarity of these EVs among the vast array of circulating particles continues to pose a considerable challenge. Accurately tracing the tissue origins of EVs depends on the presence of specific surface‐associated markers that can be targeted using affinity‐based methods (Adnan et al. 2023). However, suitable markers are often limited across different tissues, underscoring the need for rigorous characterisation and validation when using tissue‐specific EVs as biomarkers. The 2023 minimal information for studies of extracellular vesicles (MISEV) guidelines also the importance of precise nomenclature and caution against assuming the origin of EVs—whether by cell type or intracellular biogenesis pathway (Welsh et al. 2024). Even when EVs are isolated directly from organs or tumour tissues, contamination from circulating EVs via the vasculature remains a concern, potentially compromising claims of tissue specificity and marker identification (Useckaite et al. 2023).

In this manuscript, we provide a comprehensive review of current approaches to detecting and isolating tissue‐specific EVs from the blood and collate the markers proposed for various organs. We also address the inherent challenges and considerations involved in affinity capture methods, with a focus on overcoming the limitations in the current understanding of EV biology that restricts the broader application of EV‐based biomarkers. Our goal is to offer a valuable resource for researchers aiming to utilise cell‐type‐specific EVs in blood to advance the field towards truly selective liquid biopsies capable of informing both physiological and clinical insights.

## 2 EV Subtypes and Isolation

EV populations are highly diverse, with variation arising not only from differences between cells but also in the populations of vesicles that are released by a single cell under phenotypic and environmental stimuli (Dixson et al. 2023). Although EV subclasses can be generally defined by their routes of biogenesis, an evolving understanding points to extensive heterogeneity within these categories, leading to a greater number of subpopulations with distinct cargo related to their functional roles (Greening and Simpson 2018).

The two main mechanisms for the production of EVs within cells include the endosomal pathway and membrane shedding, resulting in subtypes of EVs that are broadly classified as exosomes and microvesicles (or ectosomes), respectively (Mathivanan et al. 2021). Though their size range overlaps, the limitation to exosome size (30–150 nm diameter) is dictated by their intracellular pathway. The early endosome buds inward to form many intraluminal vesicles within multivesicular bodies (MVBs). This pathway is an important cellular recycling mechanism and a key site of protein sorting, storage, transport and degradation. MVBs may divert to lysosomal degradation or fuse with the plasma membrane to release internal contents to the extracellular space; at which point the vesicles are designated exosomes (Mathivanan et al. 2021). Microvesicles form by direct budding from the plasma membrane, which is also observed for the generation of large oncosomes released by cancer cells (Minciacchi et al. 2015). This involves alteration of the local membrane curvature and a sequence of calcium influx and enzyme activation to promote the rearrangement of the actin cytoskeleton and membrane components to facilitate membrane budding and scission (Mathivanan et al. 2021). Microvesicles range in size from 100 to 1000 nm. Another class of large EVs include apoptotic bodies, which form in blebs from the plasma membrane of cells undergoing programmed cell death. Several other classes of vesicles have also been described in association with certain cell types (e.g., migrasomes), structural features (e.g., ciliary ectosomes) or cellular processes (e.g., secreted midbody remnants) (Buzas 2023; Rai et al. 2021). Proteomic‐based characterisation of EV classes has revealed common EV proteins across biological sources, proposed subtype‐specific markers and shed light on the mechanisms for sorting of cellular material into EVs formed through different pathways (Kugeratski et al. 2021; Kowal et al. 2016; Greening et al. 2017). Importantly, studies comparing the cargo profiles of EV subtypes from the same origin reveal distinct molecular composition with specific functions, underscoring the inherent heterogeneity and influence of isolation methods on EV‐derived diagnostic markers (Willms et al. 2016). For instance, mRNA profiling of exosome and ectosome subpopulations from prostate cancer cell lines demonstrated that the latter was relatively enriched in cancer‐related transcripts (Lázaro‐Ibáñez et al. 2017). Similarly, specific lipid and protein signatures were detected in small and large EV populations isolated from cultured adipocytes, with small EVs more enriched in cholesterol and proteins related to cell adhesion and macrophage activation, whilst large EVs displayed more externalised phosphatidylserine and enrichment of mitochondrial enzymes (Durcin et al. 2017). Differences between EV subtypes are also observed in biofluid sources and are related to disease (Guan et al. 2021; Bruschi et al. 2019). Proteomic analysis of exosomes and microvesicles from the urine of patients with kidney disease revealed almost 60% overlap in protein content. However, a small proportion of unique proteins were identified for each subtype, and these proteins were important for discriminating between disease states (Bruschi et al. 2019).

Given its impact on the composition of EV populations in biofluids, understanding the diversity of EV biogenesis is essential for effectively applying isolation strategies, especially when targeting specific subpopulations. EV subtypes largely overlap with one another, as well as with abundant non‐vesicular particles in the biofluid, with respect to the physical and biochemical properties typically employed for their separation (Monguió‐Tortajada et al. 2019). This presents a major challenge to EV isolation and especially complicates efforts to separate specific subtypes. Frequently used methods, including ultracentrifugation, density gradient separation, size exclusion chromatography (SEC), filter concentration and asymmetric flow‐field fractionation, typically exploit size and/or buoyant density of particles, whilst precipitation may be done by adding polymers that alter solubility (Welsh et al. 2024). Each of these approaches varies in the overall recovery and the specificity (or purity), and whilst typically more effective in simpler matrices, such as cell culture supernatants, applying them to complex biofluids like plasma and serum remains technically challenging. This complexity is exemplified by the high concentrations of lipoprotein classes in blood, which share similar size and density characteristics with EVs and outnumber them by several orders of magnitude (Simonsen 2017).

**FIGURE 1:**
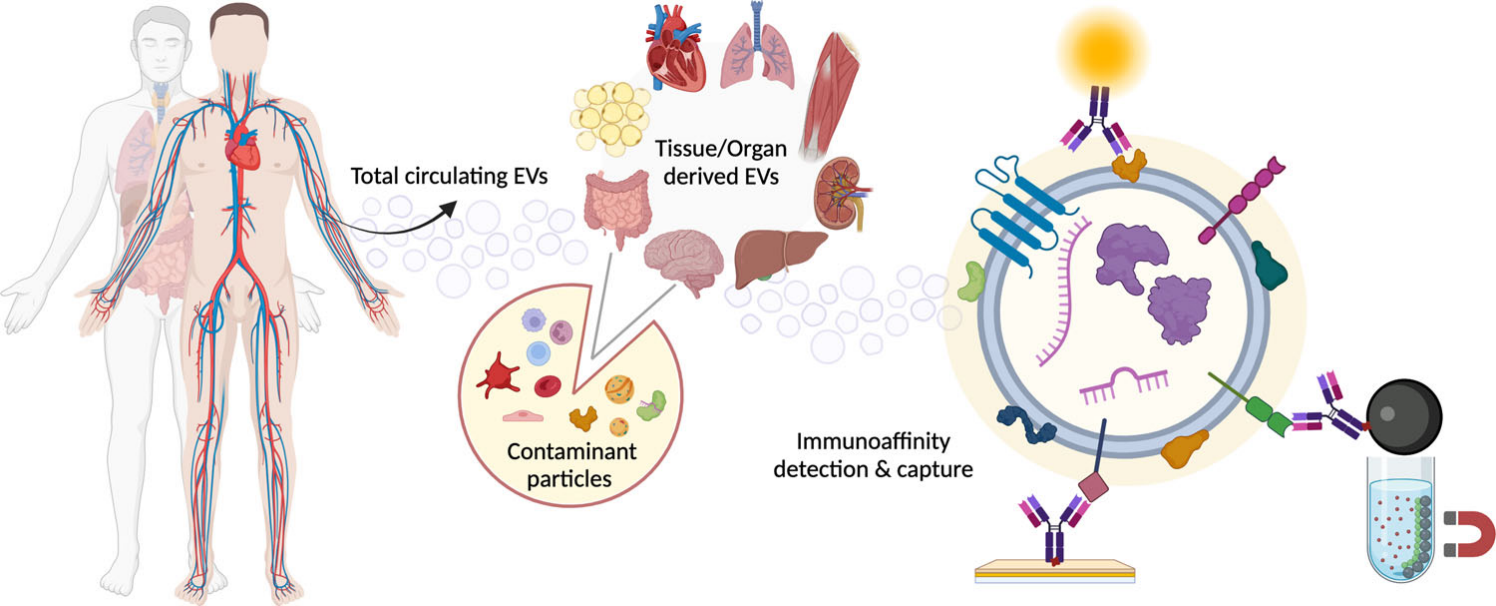
Circulating extracellular vesicles are derived from multiple origins. EVs from solid tissues represent a minor fraction among contaminant particles, including EVs from non‐target cell types, especially haematopoietic cells, albumin, lipoproteins and other abundant plasma proteins. Immunoaffinity detection or capture (IAC) methods can be used to selectively analyse target EVs based on surface marker expression. IAC confers critical benefits to liquid biopsy applications due to (i) removal of signal for a marker of interest from alternate tissue sources (enhances specificity) and (ii) greater depletion of abundant contaminants that supress signal for the marker (enhances sensitivity).

To address these issues, selective enrichment strategies are increasingly being incorporated into modern isolation platforms (Liangsupree et al. 2021). One such approach is immunoaffinity isolation, which uses affinity capture reagents—typically antibodies or other molecular probes immobilised on beads, plates or chips—to selectively pull down particles expressing specific surface markers. As these targeted strategies aim to improve subtype specificity, in this context, the purity of isolates is defined not only by the depletion of co‐isolated matrix contaminants but also by the successful recovery of vesicles from a given origin. This approach aims to enrich for subpopulations containing cargo that may otherwise be obscured by a background of abundant impurities and irrelevant subpopulations of EVs (Newman et al. 2023) (Figure 1).

## 3 Composition of Circulating EVs

The composition of circulating EVs is dynamic, governed at any given time by a balance of secretion and elimination from the blood (Auber and Svenningsen 2022). In healthy individuals, the majority of circulating EVs are believed to arise from haematopoietic cells, including platelets, erythrocytes and leukocytes, as well as endothelial cells contacting the blood (Alberro et al. 2021). Given the abundance and roles of these cell types in maintaining haematological homeostasis, this finding is physiologically intuitive. However, a recent study which integrated data on the abundance of blood cells and their EVs in circulation estimated secretion rates and EV‐to‐cell ratios, reporting that the contributions of different blood cells to circulating EV number is highly variable, ranging from 0.13 erythrocyte‐derived EVs/erythrocyte to 1.9 × 10^3^ monocyte‐derived EVs/monocyte (Auber and Svenningsen 2022). Interestingly, EV secretion in vivo does not appear to be directly related to the size, lifespan or abundance of the parental cell type, but may be associated with metabolic activity. EV secretion rates differ significantly across cell types from other tissues in vitro, and, whilst this is challenging to quantify in vivo, there likely exist cell‐type‐specific mechanisms influencing biogenesis and uptake of EVs (Auber and Svenningsen 2022; Holcar et al. 2024). Understanding circulating EV composition is further complicated by the large inter‐individual variability observed under physiological conditions, related to demographic, clinical and lifestyle factors (Holcar et al. 2024).

A variety of methods have been developed to investigate EV origins in the bloodstream, relying on different molecular cargo and presenting distinct advantages and limitations. For instance, computational deconvolution algorithms, using tissue‐specific transcriptomic signatures within circulating EVs, consistently show a predominance of blood cell derived EVs, with solid tissue contributions potentially as low as 0.2% (Li et al. 2020). However, these models are unable to account for factors such as differential sorting of RNA into EVs and variations in secretion rate across cell types, which can result in a skewed representation of EV sources in circulation. A recent study attempted to improve accuracy by creating in silico mixtures of EVs derived from different cell types within a plasma matrix to benchmark deconvolution methods (Larsen et al. 2024). Nevertheless, without a true reference for cellular compositions under native, dynamic conditions, considering fluctuations between and within individuals, accurate prediction of EV origins remains challenging. Proteomic studies have also characterised the circulating EV proteome, revealing candidate tissue‐specific proteins for EVs derived from multiple organs, including the liver, brain and adipose tissue (Rai et al. 2024; Muraoka et al. 2022; Vallejo et al. 2023). Although highly sensitive proteomic techniques, such as data independent acquisition (DIA), applied to plasma and serum EVs have identified more than 4000 unique proteins (Rai et al. 2024; Muraoka et al. 2022), still achieving sufficient depth for rarer tissue‐specific proteins requires an approach to targeted enrichment. Like transcriptome profiling, proteomic studies also constitute bulk EV analyses susceptible to similar assumptions about cargo distribution. Hence, single‐vesicle quantitative techniques, such as flow cytometry‐based detection of surface protein markers, can offer additional insights and help to refine these estimates (Holcar et al. 2024). This approach has historically focussed on EVs from blood cells (Gustafson et al. 2015; Brahmer et al. 2019; Wiklander et al. 2018; Welsh et al. 2018), whilst comprehensive immunophenotyping of tissue‐derived EVs is scarcer, owing in part to the lack of validated markers selective for tissue origins (discussed later).

It should be noted that blood EV composition will be influenced by pre‐analytical factors. Methodological differences in blood processing and EV collection between studies impact the amount of residual platelets and red blood cells and the potential for EV release ex vivo, possibly leading to overestimation of their contributions (Nieuwland and Siljander 2024). Varying estimates of different tissue contributions have been reported based on protein markers, conflicting with the abundances based on transcriptomic studies mentioned above. In healthy humans, for instance, one study suggested up to 20% may come from hepatocytes (Povero et al. 2020), 1%–5% from skeletal muscle (Guescini et al. 2015) and 1%–4% from the colon (Nazarova et al. 2021) and in pregnant women, up to 15% may arise from the placenta (Li et al. 2023). A transgenic mouse model was also used to confirm the presence of cardiomyocyte‐derived EVs in circulation, demonstrating that 3% of total EVs were positive for the fluorescent reporter (Hegyesi et al. 2022). However, these results should be interpreted with caution, given that the estimated abundance of specific subpopulations will depend on the cross‐reactivity of the antibodies used for their detection and the sensitivity and specificity for EV among non‐EV particles of the methods used to enumerate total EVs for the denominators of proportions.

In all, the composition of EVs in circulation is governed by cell‐specific secretion rates, differential biodistribution, and the impact of analytical and processing methods. Although hematopoietic cells dominate the EV landscape in blood, characterising the contributions of solid tissues will be important for gaining insights into their roles in interorgan crosstalk and the feasibility of accessing biomarkers that inform on the functional status of originating organs.

## 4 Tissue‐Derived EVs

EVs have been detected or isolated from blood plasma or serum with putative origins within multiple different organs, including the brain, liver, adipose, intestine, skeletal muscle, heart, lung and placenta (Table 1). Appreciating that organs are composed of more than one tissue type with multiple different cell types, EVs from these sources are fundamentally heterogenous; though key parenchymal cells may be targeted to represent the tissue origin (e.g., neuronal EVs from brain, hepatocyte derived EVs from the liver, etc) (Wang et al. 2025). A growing body of literature supports the notion that EVs produced within different tissues can enter the bloodstream and be accessed to understand changes in physiological and pathological conditions and provide diagnostic and predictive biomarkers (Abdelmohsen et al. 2023). Solid tissue biopsies are still used to diagnose and stage several pathologies, especially in oncology and assessing fibrosis in chronic liver diseases, as they can provide insight into morphological changes in the tissue (Tong et al. 2022; Lone et al. 2022). However, they are invasive, causing risks to patients, and are unsuited to (i) repeated sampling, (ii) sampling from sensitive tissues, such as the brain, and (iii) sampling from vulnerable patient groups, such as in pregnancy. Although traditional biopsies represent a static measurement at the time of collection, the low invasiveness—and hence repeatability—of a liquid biopsy approach offers insight into dynamic changes occurring at a molecular level over time and in response to interventions (Lin et al. 2021). Isolation methods that non‐specifically recover EVs from the blood may provide a picture of systemic bodily function, but they tend to dilute changes in specific tissues of interest. Immunoaffinity methods have thus been applied to selectively enrich EVs to monitor brain‐derived EV biomarkers in neurological disorders, liver EVs in hepatic conditions, or placental EVs in pregnancy, among many other examples (Table 1). In addition to monitoring native EVs, one group isolated EVs released from transplanted kidney, heart and islet cells, exploiting the unique human leukocyte antigen (HLA) markers on donor tissues and demonstrated the ability to monitor immunological rejection at early stages with low invasiveness (Vallabhajosyula et al. 2017; Habertheuer et al. 2018).

**TABLE 1:** Proposed markers for detection or enrichment of EVs derived from various origins from blood and other biological sources with annotation of protein expression and localisation from Human Protein Atlas and UniProt databases.

Origin	Cell type	Marker	Biofluid	Detection /enrichment method	References	Tissue expression	Subcellular localisation
BRAIN	Neuron	L1CAM	Plasma, serum; Saliva	IAC Single EV imaging Western blot	Mustapic et al. 2017; Dutta et al. 2021, Jiang et al. 2020, Jiang et al. 2020, Delgado‐Peraza et al. 2023, You et al. 2023, Shi et al. 2014, Anastasi et al. 2021, Dagur et al. 2019, Kumar et al. 2021, Goetzl et al. 2016, Fiandaca et al. 2015, Goetzl et al. 2015, Blommer et al. 2023, Bhargava et al. 2020, Delgado‐Peraza et al. 2021, Goetzl et al. 2022, Kumar et al. 2022, Mullins et al. 2017, Nasca et al. 2021, Niu et al. 2020, Shi et al. 2016, Peltz et al. 2020, Pulliam et al. 2020, Sun et al. 2017, Vreones et al. 2023, Guix et al. 2018, Banack et al. 2020, Yuan et al. 2020, Zou et al. 2020, Mansur et al. 2020, Madhu et al. 2019, Athauda et al. 2019, Sun et al. 2019, Katsu et al. 2019, Kapogiannis et al. 2019, Agliardi et al. 2019, Si et al. 2019, Cha et al. 2019, Winston et al. 2019, Kapogiannis et al. 2015, Goetzl et al. 2020, Chawla et al. 2019, Kapogiannis et al. 2019, Zhao et al. 2019, Winston et al. 2018, Patterson et al. 2018, Goetzl et al. 2019, Gill et al. 2018, Goetzl et al. 2015, Edwardson et al. 2024, Lee et al. 2020 Nogueras‐Ortiz et al. 2024 Rani et al. 2019	CNS, PNS, distal renal tubules, melanocytes, endothelial cells	Single pass type 1 membrane protein, cytoplasmic, secreted
		NCAM1	Plasma, tissue, CSF	IAC MS ECL	You et al. 2023, Fiandaca et al. 2015, Kumar et al. 2022 Kapogiannis et al. 2015, You et al. 2022, Jia et al. 2019 Huang et al. 2022	CNS, PNS, adrenal gland, heart, gastric chief cells	Single pass type 1 membrane protein, cytoplasmic, secreted
		ATP1A3	Plasma, tissue, CSF	IAC MS	You et al. 2023 You et al. 2022	CNS, cardiac myocytes	Multi‐pass membrane protein
		SYP	Plasma; tissue	IAC MS, single vesicle imaging	Kumar et al. 2022 Chand et al. 2020	CNS, islets of Langerhans, peripheral nerves, enteroendocrine cells	Multi‐pass membrane protein
		GAP43	Plasma	IAC	Eitan et al. 2023	CNS, PNS	Peripheral membrane protein
		NLGN3	Plasma	IAC	Eitan et al. 2023	CNS	Single pass type 1 membrane protein
		DPP6	Tissue, serum	MS, western blot	Abdelmohsen et al. 2023	CNS, smooth muscle	Single‐pass type 2 membrane protein
		SYT1	Tissue, serum	MS, western blot	Abdelmohsen et al. 2023	CNS	Single‐pass membrane protein
		DNM1L	Tissue, serum	MS, western blot	Abdelmohsen et al. 2023	Expression in several tissues	Peripheral membrane protein
		SNAP25	Plasma	ELISA	Ohmichi et al. 2019	CNS	Membrane associated
		STXBP1	Plasma, serum	MS	Muraoka et al. 2022	Selective expression in CNS, PNS and pancreatic islets	Peripheral membrane protein
		GPM6A	Plasma, serum	MS	Muraoka et al. 2022	CNS	Multi‐pass membrane protein
		PSD2	Plasma, serum	MS	Muraoka et al. 2022	CNS	Single‐pass membrane protein
		GDI1	Plasma, serum	MS	Muraoka et al. 2022	General cytoplasmic expression	Cytoplasm
	CSPG4‐type neurons	CSPG4	Plasma	IAC	Goetzl et al. 2019	Ubiquitous granular cytoplasmic expression	Single‐pass type 1 membrane protein
	Oligodendrocyte	MOG	Plasma, serum	IAC	Dutta et al. 2021, Edwardson et al. 2024, Agliardi et al. 2023	CNS (selective oligodendrocytes)	Single‐pass type 1 or multi‐pass membrane protein
		OMgp	Plasma, serum	IAC ELISA	Agliardi et al. 2024 Ohmichi et al. 2019	CNS (selective oligodendrocytes)	Membrane associated
		CNP	Plasma, serum	IAC MS	Yu et al. 2020 Muraoka et al. 2022	Oligodendrocytes, peripheral nerves	Membrane associated
		SLC44A1	Plasma, serum	MS	Muraoka et al. 2022	Ubiquitous expression	Multi‐pass membrane protein
		PDGFRα	Plasma	IAC	Kumar et al. 2023	Enterocytes, glomerulus, myoepithelial cells, endometrium	Single‐pass type 1 membrane protein
		LAMP2	Tissue	MS	You et al. 2022	Ubiquitous granular cytoplasmic expression	Single‐pass type 1 membrane protein
		FTH1	Tissue	MS	You et al. 2022	General cytoplasmic expression	Cytoplasm
	Astrocyte	GLAST	Plasma, serum Tissue	IAC MS ELISA	Kumar et al. 2021, Bhargava et al. 2020, Delgado‐Peraza et al. 2021, Madhu et al. 2019, Winston et al. 2019, Edwardson et al. 2024, Lee et al. 2020, You et al. 2022, Kumar et al. 2023, Goetzl et al. 2018, Goetzl et al. 2018, Goetzl et al. 2016, Valle‐Tamayo et al. 2022 Ohmichi et al. 2019	CNS (astrocytes), retina	Multi‐pass membrane protein
		EAAT2	Plasma tissue	Flow cytometry IAC	Zhang et al. 2024, D'Ambrosio et al. 2024	CNS (astrocytes)	Multi‐pass membrane protein
		LRP1	Tissue	IAC, MS	You et al. 2022	Expressed in several tissues	Single‐pass type 1 or peripheral membrane protein, cytoplasm
		GFAP	Plasma	Western blot	Kodidela et al. 2020	CNS (astrocytes)	Cytoplasm
	Microglia	TMEM119	Plasma tissue	IAC ECL	Kumar et al. 2021, Kumar et al. 2023, Roseborough et al. 2023 Huang et al. 2022	Highly expressed in microglia, medium expression in several cell types	Single‐pass type 1 membrane protein, ER membrane, cytoplasmic, secreted
		CX3CR1	Plasma	Flow cytometry	Duan et al. 2024	Expression in several tissues	Multi‐pass membrane protein
		UCHL1	Plasma	Flow cytometry	Duan et al. 2024	Selective expression in CNS, distal renal tubules, islets of Langerhans and spermatogonia in testis	Membrane associated
		ITGAM	Tissue	MS	You et al. 2022	Selective cytoplasmic expression in subset of immune cells and glial cells	Single‐pass type 1 membrane protein
		LCP1	Tissue	MS	You et al. 2022	Selective cytoplasmic expression in immune cells	Peripheral membrane protein, cytoplasm
LIVER	Hepatocyte	ASGR1	Plasma, serum, tissue	IAC MS Flow cytometry	Rodrigues et al. 2021, Rodrigues et al. 2022, Newman et al. 2022 Abdelmohsen et al. 2023, Newman et al. 2021 Povero et al. 2020, Newman et al. 2021, Li et al. 2019, Rega‐Kaun et al. 2019	Distinct expression in hepatocytes, low expression in stomach	Single‐pass type 2 membrane protein, secreted
							
		ASGR2	Plasma tissue	nPES MS	Nakao et al. 2021, Sehrawat et al. 2021 Abdelmohsen et al. 2023	Selective expression in hepatocytes	Single‐pass type 2 membrane protein, secreted
		CYP2E1	Plasma, serum	nPES Flow cytometry	Nakao et al. 2021, Sehrawat et al. 2021 Li et al. 2019	Selective expression in hepatocytes	Peripheral membrane protein, ER associated
		SLC27A5	Plasma, serum	Flow cytometry	Povero et al. 2020	Selective expression in hepatocytes	Multi‐pass membrane protein
		ARG1	Serum tissue	ELISA MS	Li et al. 2022 Abdelmohsen et al. 2023	Selective expression in liver and subsets of bone marrow cells	Cytoplasm
		FABPL	Tissue	MS, single vesicle imaging	Abdelmohsen et al. 2023, Chand et al. 2020	Liver, intestinal glands and renal tubules	Cytoplasm
ADIPOSE	White adipocytes	ADIPOQ	Plasma serum, tissue	Western blot, ELISA MS, immunogold labelling	Blandin et al. 2023, Connolly et al. 2018, Phoonsawat et al. 2014 Garcia‐Martin et al. 2022	Selective expression in adipocytes	Secreted
		PLIN1	Plasma	Western blot	Eguchi et al. 2016, Connolly et al. 2018, Kobayashi et al. 2018	Selective expression in adipocytes	ER membrane and lipid droplet surface associated
		PPARG	Plasma	Western blot	Connolly et al. 2018	Expression in several tissues	Nucleus, cytoplasm
		FABP4	Plasma, serum	IAC Western blot Flow cytometry	Hubal et al. 2017, Mishra et al. 2023, Siqueira et al. 2022 Connolly et al. 2018 Gustafson et al. 2015	Selective expression in adipocytes	Nucleus, cytoplasm
		CA3	Plasma	IAC	Mishra et al. 2023	Selective expression in adipocytes and skeletal muscle cells	Cytoplasm
		STEAP4	Plasma	IAC	Mishra et al. 2023	Enhanced in adipocytes	Multi‐pass membrane protein
		GGT5	Plasma	IAC	Mishra et al. 2023	Expression in adipocytes, stromal and endothelial cells	Single‐pass type 2 membrane protein
		CAMK2A	Plasma	IAC	Mishra et al. 2023	Ubiquitous expression	Membrane associated
		PREF‐1	Plasma	Flow cytometry	Gustafson et al. 2015	Expression in adrenal gland, islets of Langerhans and stroma cells in placenta	Single‐pass type 1 membrane protein
		GDN	Tissue, serum	MS, western blot	Abdelmohsen et al. 2023	Expressed in placenta, ovary	Secreted
	Brown adipocytes	POSTN	Serum, tissue	MS, immunogold labelling	Garcia‐Martin et al. 2022	Ubiquitous expression	Secreted
INTESTINE	Intestinal epithelial cells	GPA33	Plasma, CCM	IAC	Nazarova et al. 2021, Gotanda et al. 2016, Mathivanan et al. 2010	Selective expression in intestine	Single‐pass type 1 membrane protein
		CLRN3	Plasma	IAC	Nazarova et al. 2021	Expression in intestinal tract, kidney, liver and gallbladder	Multi‐pass membrane protein
		GCNT3	Plasma	IAC	Nazarova et al. 2021	Expression in gastrointestinal mucosa, kidney	Single‐pass type 2 membrane protein
		PIGY	Plasma	IAC	Nazarova et al. 2021	Low tissue specificity	Multi‐pass membrane protein, ER associated
		REG4	Plasma	IAC	Nazarova et al. 2021	Selective expression in goblet cells	Secreted
		ACE	Plasma, serum	MS	Muraoka et al. 2022	Expression in lung capillaries, small intestine, renal tubules and male genitalia	Single‐pass type 1 membrane protein, cytoplasm, secreted
		PLCB3	Plasma, serum	MS	Muraoka et al. 2022	Selective expression in glandular cells of small intestine and Purkinje cells in cerebellum	Cytoplasm
SKELETAL MUSCLE	Skeletal myocytes	SGCA	Plasma	IAC Flow cytometry	Guescini et al. 2015, Fulzele et al. 2019 Conkright et al. 2022, Conkright et al. 2022, Maggio et al. 2023	Expression in several tissues, most prominent in sarcolemma	Single‐pass type 1 membrane protein
		SPARC	Serum, tissue	MS, immunogold labelling	Garcia‐Martin et al. 2022	Expression in fibroblasts, endothelial cells, megakaryocytes, subsets of glial cells, and in testis seminiferous ducts	Secreted
		ATP2A1	Plasma	Western blot	Watanabe et al. 2022	Selective expression in skeletal muscle	Multi‐pass membrane protein, endoplasmic and sarcoplasmic reticulum associated
		ENO3	Plasma	Western blot	Watanabe et al. 2022	Selective expression in skeletal muscle	Cytoplasm
		DES	Plasma	Western blot	Watanabe et al. 2022	Expression in heart, skeletal and smooth muscle	Cytoplasm, sarcolemma membrane associated
		XIRP2	Plasma, serum	MS	Muraoka et al. 2022	Expression in heart and skeletal muscle	Cell junctions
		STK25	Plasma, serum	MS	Muraoka et al. 2022	Expression in several tissues	Cytoplasm, golgi apparatus
		ALDOA	Plasma, serum	MS	Muraoka et al. 2022	Expression in most cell types, enhanced in skeletal myocytes	Cytoplasm
		PKM	Plasma, serum	MS	Muraoka et al. 2022	Expression in most tissues	Cytoplasm
		PGM1	Plasma, serum	MS	Muraoka et al. 2022	Expression in several tissues, enhanced in hepatocytes	Cytoplasm
		RAD23A	Plasma, serum	MS	Muraoka et al. 2022	Ubiquitous expression	Nucleus
HEART	Cardiomyocytes	LDB3	Plasma, tissue	MS	Abou Zeid et al. 2022	Expression in heart, skeletal muscle	Cytoplasm
		CAV3	Tissue, serum	MS, single vesicle imaging	Chand et al. 2020	Expression in heart, skeletal muscle	Peripheral membrane protein
		CPT1B	Tissue, serum	MS, western blot	Abdelmohsen et al. 2023	Expression in heart, skeletal muscle, parathyroid gland and testis	Multi‐pass membrane protein (outer mitochondrial membrane)
		CX43	Plasma	Flow cytometry	Rodriguez et al. 2018	Ubiquitous expression	Multi‐pass membrane protein, gap junction, ER associated
		CD172a	Serum Plasma	Peptide probe + AgNP electrochemical detection Flow cytometry	Zhou et al. 2023 Anselmo et al. 2021	Ubiquitous expression	Single‐pass type 1 membrane protein
		MYH6	Whole heart perfusate (mouse)	MS	Claridge et al. 2021	Expression in heart and skeletal muscle	Cytoplasm
		ACTN1	Whole heart perfusate (mouse); Plasma, serum	MS	Claridge et al. 2021 Muraoka et al. 2022	Ubiquitous expression	Cytoplasm
	Cardiac fibroblasts	CKAP4	Whole heart perfusate (mouse); Plasma, serum	MS	Claridge et al. 2021 Muraoka et al. 2022	Ubiquitous expression	Single‐pass type 2 membrane protein, ER associated, cytoplasm
LUNG	Bronchiolar Clara cells, alveolar type 2 cells	CCSP	Exhaled breath condensate	IAC	Mitchell et al. 2024	Selective expression in subset of respiratory epithelial cells in bronchus	Secreted
		SFTPC	Exhaled breath condensate	IAC	Mitchell et al. 2024	Selective expression in pneumocytes	Secreted
		SFTPA	Plasma Tissue, serum	EVarray (ELISA) MS, western blot	Jakobsen et al. 2015 Abdelmohsen et al. 2023	Highly specific expression in type II pneumocytes	Secreted
		SFTPB	Tissue; plasma, serum	MS, single vesicle imaging	Chand et al. 2020 Muraoka et al. 2022	Selective expression in pneumocytes	Secreted
		SFTPD	Plasma	EVarray (ELISA)	Jakobsen et al. 2015	Selective expression in pneumocytes	Secreted
PLACENTA	Syncytiotrophoblasts	PLAP	Plasma	IAC Flow cytometry ELISA	Mitchell et al. 2024, Sabapatha et al. 2006, Kandzija et al. 2019, Shinde et al. 2024, Rao et al. 2024 Li et al. 2023, Dragovic et al. 2013, Orozco et al. 2009, Tersigni et al. 2021 Sarker et al. 2014, Salomon et al. 2017 Pillay et al. 2016	Specific expression in placental trophoblasts	Membrane associated
		ERVW‐1	Plasma, serum	Western blot, Qdot ELISA	Levine et al. 2020 Vargas et al. 2014	Specific expression in placental trophoblasts	Single‐pass type 1 membrane protein
		ERVW‐2	Serum	ELISA	Vargas et al. 2014	Specific expression in placental trophoblasts	Single‐pass type 1 membrane protein
	Extravillous trophoblasts	HLA‐G	Plasma, serum	IAC Flow cytometry MS	Shinde et al. 2024, Rao et al. 2024 Orozco et al. 2009 Muraoka et al. 2022	Distinct expression in extravillous trophoblasts	Single‐pass type 1 membrane protein, endosomal, secreted
KIDNEY	Renal parenchymal cells	SLC3A1	Tissue, serum	MS, western blot	Abdelmohsen et al. 2023	Highly expressed in kidney and intestine	Single‐pass type 2 membrane protein
		SLC22A2	Tissue	MS, single vesicle imaging	Chand et al. 2020	Specific expression in renal tubules	Multi‐pass membrane protein
	Mesangial cells	ITGA8	Plasma	Flow cytometry	Komatsu et al. 2024	Specific expression in glomerulus	Single‐pass type 1 membrane protein

Abbreviations: ECL, electrochemiluminescence‐linked immunoassay; ELISA, enzyme‐linked immunosorbent assay; IAC, immunoaffinity capture; MS, mass spectrometry; nPES, nanoplasmonic enhanced scattering.

Table 1 provides a summary of studies reporting the detection or isolation of EVs from specific origins and the candidate markers are given for specific cell types within the various tissues. There is a focus on studies from plasma or serum as the biological source, given the blood is the most common source reported for EV liquid biopsies and vascularisation provides a window into virtually all organ sources (Nieuwland and Siljander 2024). For some tissues, there is a significantly larger body of research, especially the brain and adipose tissue, whilst for others, only a limited number of candidate markers have been proposed and investigated. Although renal‐derived EVs have been examined in the blood (Abdelmohsen et al. 2023; Komatsu et al. 2024), the more frequent source material is urine, given the majority of urinary EVs derive from the urogenital tract (Erdbrügger et al. 2021). The origins of urinary EVs are beyond the scope of this review, but similarly to blood EVs, cell‐type specific markers have been proposed and one study using flow cytometry on minimally processed urine samples, quantified EVs derived from 10 renal cell types based on the expression of 18 different surface markers (Turco et al. 2016). Candidate markers have been included from studies which extracted EVs directly from tissues and screened by untargeted mass spectrometry (MS)‐based proteomics (Abdelmohsen et al. 2023; Garcia‐Martin et al. 2022; Chand et al. 2020). Strengths of these studies included the comparison of multiple tissues to provide evidence of selectivity to the organ and subsequently demonstrating the ability to detect many of the markers in EVs isolated from the circulation.

### 4.1 Detection Versus Separation

EVs can be analysed either directly in biofluids or after a separation or enrichment method is applied (Welsh et al. 2024). This distinction is critical when aiming to assess tissue origins of EVs or examine the content of subpopulations. The presence and abundance of EVs from certain origins can be measured based on given markers within the biofluid or the EV preparation, whereas enrichment methods, such as IAC, separate them from the total EV‐containing material and permit subsequent analysis of subpopulation‐specific cargo. Like IAC, some methods for detecting tissue‐derived EVs rely on markers present on the surface, including flow cytometry, imaging techniques or ELISA, whilst intraluminal protein markers can only be detected using methods that first disrupt EV membranes (Shah et al. 2018) (e.g., western blotting, MS). Although detecting tissue‐derived EVs in non‐enriched samples has shown biomarker potential for assessing the liver (Povero et al. 2020; Nakao et al. 2021; Sehrawat et al. 2021), adipose tissue (Gustafson et al. 2015; Eguchi et al. 2016) and placental health (Li et al. 2023), these approaches inherently limit the number and types of molecular markers that can be analysed whilst preserving specificity for their tissue origin. Since the EV subpopulation of interest likely contains other condition‐associated molecular cargo, selectively extracting EVs based on a suitable marker allows this additional information to be analysed in a specific manner, regardless of its expression elsewhere in the body (Newman et al. 2022). For instance, cytochrome P450 (CYP) enzymes are important mediators of xenobiotic metabolism with expression in multiple tissues that can be differentially induced and inhibited by co‐administered agents (Newman et al. 2024). Our group has recovered hepatocyte‐specific EVs from blood to determine the pattern of CYP protein induction occurring in the liver (Rodrigues et al. 2021; Rodrigues et al. 2022), whilst another group similarly measured miRNA in intestine‐specific EVs reflecting intestinal activity of the breast cancer resistance protein (BCRP) (Gotanda et al. 2016). In each case, abundance of the molecular cargo in tissue‐specific EVs, but not total circulating EVs, could accurately predict exposure to the respective substrate medicines of the enzyme. Across multiple tissues, immunoaffinity capture (IAC) has been effectively coupled to downstream molecular analyses to understand disease‐associated changes in the tissue molecular profile from the blood. For example, transcript and metabolite cargo in circulating adipocyte EVs pre and post gastric bypass surgery reflect changes in glucose homeostasis and insulin resistance (Hubal et al. 2017), alpha‐synuclein content in neuronal or oligodendrocyte derived EVs can distinguish Parkinson's disease and multiple system atrophy (Dutta et al. 2021; Jiang et al. 2020; Jiang et al. 2020; Ohmichi et al. 2019; Yu et al. 2020), various neuroprotective proteins in plasma neuronal EVs differ in mild and moderate Alzheimer's disease and in response to exercise (Delgado‐Peraza et al. 2023), and we reported the capacity for miRNA markers in liver derived EVs to reflect the severity of non‐alcoholic fatty liver disease (Newman et al. 2022).

### 4.2 Specificity of Protein Expression

Using the Human Protein Atlas (HPA) database (http://www.proteinatlas.org), which reports expression profile in tissues on both mRNA and protein level based on RNA sequencing (RNAseq) data, immunohistochemistry and knowledge‐based annotation, the collection of candidate markers was cross‐referenced for tissue‐specificity. This supported selective expression of ten brain markers across three cell types, three markers each of hepatocytes and adipocytes, two each of intestinal, kidney and skeletal muscle cells, five lung markers, and four markers of placenta tissue across two cell types (Table 1). Evaluation of markers is challenged by the discordance between transcripts and protein in terms of their tissue enrichment or specificity (Jiang et al. 2020). Muraoka et al. (2022) reported a catalogue of proteins detected by DIA MS from plasma and serum EVs including markers for several tissues, but noted fewer tissue‐specific proteins based on tissue proteome datasets compared to the HPA RNAseq annotation. For example, Xin actin binding repeat containing 2 (XIRP2) mRNA is highly enriched in skeletal muscle but observed in high protein abundance in heart muscle, and PLCB3 mRNA is highly enriched in the intestine but displays moderate protein abundance across several tissues (Muraoka et al. 2022). Similarly, STEAP4 metalloreductase RNA is selectively enhanced in adipocytes but protein information is limited (Mishra et al. 2023). Though the enzyme is known to play important roles in a number of metabolically active cells (Scarl et al. 2017), it has been used as an IAC target to recover adipocyte‐EVs from plasma (Mishra et al. 2023). Selecting markers based on evidence at RNA level, thus warrants caution and careful validation incorporating information from these databases with empirical observations in EVs.

To date, the most mature body of work regarding tissue‐specific EV isolation comprises studies in EVs of putative neuronal origin, based on IAC against L1 cell adhesion molecule (L1CAM). However, despite multiple reports of biomarker signatures in L1CAM+ EVs correlating with neurological disorders, questions have been raised regarding the true selectivity of this marker. Given its expression in peripheral nerves, melanocytes and immune cells, researchers have been prompted to propose alternative markers (Nogueras‐Ortiz et al. 2024; Gomes and Witwer 2022; Norman et al. 2021). You et al. (2023) found that ATPase Na+/K+ transporting subunit alpha 3 (ATP1A3) was significantly enriched in EVs from brain tissue and plasma relative to L1CAM and neural cell adhesion molecule 1 (NCAM1), and IAC of plasma ATP1A3+ EVs co‐enriched synaptic markers and proteins related to Alzheimer's disease. Interestingly, differing sorting mechanisms between cell types was demonstrated by a comparative proteomic analysis of EVs and their originating white and brown adipocytes, hepatocytes, skeletal myocytes and endothelial cells, suggesting specific patterns of EV export that could allow their origin to be delineated from other potential sources of the marker (Garcia‐Martin et al. 2022).

### 4.3 Subcellular and Vesicular Localisation

Concerns regarding the use of L1CAM extend to the production of multiple isoforms by alternative splicing and cleavage of the ectodomain, resulting in transmembrane and soluble forms of the protein (Norman et al. 2021). L1CAM can largely be detected as extracellular proteolytic fragments in circulation, although a minor fraction of membrane‐associated L1CAM was confirmed as EV cargo in early SEC fractions (Nogueras‐Ortiz et al. 2024). Some proteins exhibit tissue‐specific patterns of isoform expression which may be leveraged to support their origin (Wang et al. 2008), for instance, neurons predominantly express full length L1CAM (Nogueras‐Ortiz et al. 2024); however, this relies on availability of antibodies to unique epitopes. The debated use of L1CAM as a marker for neuronal EVs was recently reviewed in depth by Gomes and Witwer (2022) and highlights opportunities to progress cell‐type‐specific EV isolation and analysis with appropriate validation and characterisation. Transmembrane proteins are ideal handles for IAC, as this increases confidence in the capture of intact vesicles from the biofluid. Just over half of the eighty‐five markers identified here are reported to be single‐ or multi‐pass membrane spanning, according to subcellular localisation given in the HPA and UniProt databases (The UniProt C 2021). Several were membrane‐associated or localised to other subcellular compartments, including endoplasmic reticulum, Golgi apparatus, sarcoplasmic reticulum, lipid droplets or nuclei, which will impact their incorporation into EVs produced through different biogenesis pathways. Interestingly, markers that have been used as IAC targets are not restricted to transmembrane proteins. Two secreted proteins, Clara Cell Secretory Protein (CCSP) and surfactant protein C (SFTPC), were used to isolate lung‐derived EVs in exhaled breath condensate by the “EV‐CATCHER” device (Mitchell et al. 2024). Additionally, fatty acid binding protein 4 (FABP4) has been reported to have cytoplasmic expression in adipocytes, but surface shaving coupled to MS‐proteomic analysis of plasma EVs revealed external localisation and accessibility to IAC of intact EVs (Mishra et al. 2023). This underscores persistent gaps in understanding of how protein cargo is sorted into EVs, and that localisation may not recapitulate what is observed in cells. Sorting of some transmembrane proteins into EVs has even been observed to invert their typical orientation, resulting in the ectodomain pointing into the lumen (Cvjetkovic et al. 2016). Proteins that lack a transmembrane region may also be associated with external EV membranes where they can elicit functional roles in cell signalling (Buzás et al. 2018). For example, adiponectin was shown to be localised on the surface of adipocyte‐derived EVs by immunogold labelling and capable of binding its receptors to relay insulin‐sensitising and anti‐inflammatory signals in vivo (Blandin et al. 2023). However, the authors recommended against its use as an IAC handle, given the possibility that soluble adiponectin may be nonspecifically acquired by the surface of EVs from other sources in the circulation, confounding identification of tissue origin. For other secreted proteins suggested as specific surface markers, especially those less abundant in circulation than adiponectin, questions arise about (i) whether the EV acquired the protein during its formation inside the cell or after release into a local tissue environment rich in cell‐specific proteins, (ii) the capacity of different EVs to adsorb proteins in the interstitium and circulation, and (iii) the impact of isolation methods on the identification of EV associated protein cargo (Ramos et al. 2022; Tóth et al. 2021).

Cell‐type or tissue derived EVs have been identified in blood and other biofluids using a variety of methods and marker proteins, shedding light on the role of EV subpopulations as signalling mediators and potential biomarkers of disease. However, characteristics of the marker protein, such as its selectivity of expression particularly on the protein level and its localisation with EV membranes, should be closely examined and validated. This curated list of markers could assist with selection and validation of IAC targets. For example, selectivity could be investigated using supplementary markers of the target cell type in the captured fractions and exclusion of markers of different tissues in captured fractions relative to the flow through (Welsh et al. 2024).

## 5 Tumour‐Derived EVs

Just as EVs released from various solid tissues are rare in the circulation, tumour‐derived EVs also represent a needle in the haystack of healthy cell‐derived EVs and resolving these is appealing to enhance the sensitivity and specificity of biomarkers in multiple cancers. IAC techniques have been applied to selectively extract chondroitin sulphate proteoglycan 4 (CSPG4)+ EVs from melanoma patient plasma using an antibody to the melanocyte‐specific proteoform, revealing immunoregulatory cargo and markers of progression (Sharma et al. 2018; Sharma et al. 2020); whilst prostate cancer derived EVs display prostate‐specific membrane antigen (PMSA) that can be targeted for isolation from cell culture supernatant and patient plasma (Mizutani et al. 2014; Allelein et al. 2021; Wang et al. 2023). Whereas for organ‐derived EV isolation, often a single marker was used as an IAC handle, with few exceptions including the CCSP/SFTPC double‐positive lung‐specific EV‐CATCHER platform (Mitchell et al. 2024) and a five‐strong cocktail of antibody‐conjugated beads for adipocyte‐EV capture (Mishra et al. 2023), most studies report the capture of tumour‐derived EVs using multiple markers. There is no universal specific tumour marker, although some generic proteins tend to have upregulated expression in EVs from cancer cells. Cell‐type‐specific markers may be useful for cancers in particular organs, but will not distinguish the EV from healthy cell‐derived EVs, and these may be absent from vesicles released by certain tumour types, depending on the stage of differentiation (Nazarova et al. 2021). For instance, a recent study developed a capture method for glioblastoma (GBM) derived EVs in patient circulation using panel of markers that not only target highly expressed proteins in normal brain and GBM tissue (ATP1B2 and EAAT2), but also markers of glioma stem cells in different cell‐like states (including CD24, CD44, CD133 and EGFR) (Zhang et al. 2024). The GBM‐specific EV isolation thus captured the diversity of releasing cells and their EVs in the tumour and integration of the platform with molecular profiling by surface enhanced Raman spectroscopy, demonstrated promising capability to non‐invasively reflect GBM tumour burden and response to treatments. Another study also captured circulating GBM‐derived EVs by surface expression of only one protein, tenascin C (TNC) (Salviano‐Silva et al. 2025). Despite broad systemic expression of this protein, TNC was significantly upregulated in GBM tissue, especially malignant cell populations of mesenchymal‐like phenotypes and harbouring chromosomal abnormalities. Circulating TNC+ EVs successfully distinguished GBM and healthy donors, and, with mutant alleles more frequently detected in TNC+ subpopulations than TNC‐ EVs, IAC was critical to improving the sensitivity of tumour‐derived DNA analysis.

Microfluidic devices with multiple antigen binding capacity have also been developed for rapid enrichment of EVs from the blood, and often include epithelial cell adhesion molecule (EpCAM) along with various other surface proteins shown to be upregulated in different tumours. For example, along with anti‐EpCAM binding the “EV Click‐Chips” device was adapted for diagnostic miRNA profiling of hepatocellular carcinoma EVs expressing ASGR1 and CD147 (Sun et al. 2020), and for prostate cancer using PSMA (Wang et al. 2023). Similarly, a diagnostic panel of 11 miRNAs was developed for pancreatic cancer using EVs enriched for EpCAM, CD104, CD44v6, MET and tetraspanin‐8 (Ko et al. 2018). Plasma and serum EVs from GBM multiforme patients were also enriched via a suite of tumour‐associated antigens, including wildtype and mutant variants of epidermal growth factor receptor (EGFR/EGFRvIII), platelet‐derived growth factor receptor (PDGFR), podoplanin and ephrin‐A receptor type 2 (EphA2) (Reátegui et al. 2018). The approach captured tumour heterogeneity, with RNA sequencing identifying hallmark transcripts for the different genetic subtypes of the disease. Comprehensive characterisation of the distinct class of EVs specifically released by cancer cells, known as large oncosomes, recently revealed candidate surface markers, which are expected to facilitate their specific isolation from patient biofluids with potential relevance across multiple cancer types, including breast, prostate and gliomas (Silva et al. 2024). Given that there are multiple cell populations other than the tumour that release EVs to modulate the tumour microenvironment (Tao and Guo 2020), the EV enrichment approach should consider the possible loss of relevant diagnostic information with increasing specificity of isolation.

Although this review presents studies that achieved affinity capture using antibody targeting of surface protein epitopes, the recognition of target molecules may also be achieved with aptamers. Aptamers are oligonucleotides with unique tertiary structures generated through an in vitro selection process and are capable of capturing targets with high affinity and specificity (Zhang et al. 2022). Similarly to antibodies, they can be modified or functionalised for immobilisation on solid interfaces, and exhibit excellent stability and binding kinetics, with their smaller size less prone than antibodies to steric hindrance due to the dense biomolecular corona acquired by EVs in circulation (Chowdhury et al. 2024). For these reasons, aptamers are increasingly gaining attention for EV sensing and isolation from biofluids for applications in cancer diagnostics (Zhu et al. 2023; Zhou et al. 2022; Cha et al. 2023).

## 6 Methodological Considerations for IAC

### 6.1 EV Pre‐Enrichment

Protocols for IAC of EVs from different tissue sources will either include an EV pre‐enrichment step or apply minimally processed biofluids directly to the antibody‐functionalised capture material. There are advantages and drawbacks of each approach, which should take into account the concentration of the target and performance of the antibody, weighed up against the need for throughput and reduced processing time (Fortunato et al. 2022). The composition of plasma and serum is complex and highly viscous, causing matrix effects and non‐specific binding, both to the antibody and the surface of the solid phase. In particular, the non‐specific adherence of lipids, whether in off‐target EV membranes or abundant lipoprotein particles, can distort analyses of molecular cargo (Adnan et al. 2023). Lipoproteins also interact with EVs and can contribute to the density of the EV corona—the layers of molecules that associate with the EV exterior during their formation and upon release and interaction with the extracellular milieu (Buzas 2022)—which has the potential to obscure target epitopes and reduce the efficacy of immunocapture (Hallal et al. 2022). Applying a global EV enrichment step prior to IAC can thus help to separate target EV subtypes from lipoprotein particles and decrease interference. However, increasing sample handling potentially increases EV losses and creates a trade‐off between yield and purity, and this should be considered in the choice to pre‐enrich EVs or perform IAC directly from the biofluid (i.e., unprocessed plasma or serum). Given that increasing sample handling potentially increases EV losses, however, the choice to pre‐enrich EVs or perform IAC directly in a biofluid can create a trade‐off between yield and purity. Ideally, a pre‐enrichment method should be gentle and result in sufficient purity itself in order to be useful, as excessive force or crude separation techniques, including ultracentrifugation or resin precipitation (e.g., ExoQuick), alter the native morphology and surface composition of the vesicles (Monguió‐Tortajada et al. 2019). SEC is favourable to precede IAC for several reasons; (i) it excludes up to 99% of soluble proteins from EV‐enriched fractions, which can help to support the EV association of a surface marker in cases of secreted or cleaved proteoforms, (ii) does not cause aggregation of EVs and (iii) may remove loosely associated corona proteins. However, any isolation strategy introduces bias to recovery of EV subpopulations, affecting the final composition of tissue‐derived EVs available to IAC, and so, the method should be optimised with this purpose in mind—for instance, in the case of SEC, ensuring collection of fractions that are enriched for target subpopulations.

### 6.2 Blood Processing

Pre‐analytical variables and methods for blood processing can also influence access to target EVs (Useckaite et al. 2021). Typical centrifugation protocols for obtaining plasma or serum are designed to minimise residual platelets but are likely to also remove large EVs (Nieuwland and Siljander 2024). EV subpopulations interact with the extracellular environment in different ways (Buzás et al. 2018), including aggregation, which could cause them to co‐sediment with cells during plasma and serum preparation.

### 6.3 Antibody Specifics

Together with appropriate surface marker selection, the robustness of an IAC protocol ultimately depends on antibodies that bind the intended target with high affinity and specificity. Commercially available antibodies often lack detailed validation of their cross‐reactivity with structurally similar proteins, placing the onus on researchers to carefully verify the performance of the antibody in an IAC method. To this point, the L1CAM antibodies used to enrich circulating neuronal EVs were found to also capture soluble α‐synuclein, confounding its reported utility as a brain‐specific EV‐associated biomarker (Norman et al. 2021). In addition to ensuring complete and transparent reporting of antibody characteristics (clone, isotype, epitope, etc.), useful controls in method development include isotype control antibodies, antigen‐negative samples (i.e., non‐target tissue or cell lines), and comparisons with antibodies against alternative epitopes in other domains of the protein, such as predicted internal regions (Gomes and Witwer 2022). Reagent concentrations should be optimised to maintain specificity and ensure condition‐related differences or biological variability in target EV abundance are accurately reflected in captured populations. This can be done by titrating the antibody concentration relative to the binding capacity of the solid phase, minimising exposed surfaces that could lead to non‐specific binding. Balancing the ratio of capture reagents to target entities is crucial for specificity, as reagents that lack complete selectivity will have a greater potential for off‐target capture when present in excess. Additionally, capture reagents should not be saturated with EVs to avoid equalising target abundances between samples and diminishing the capacity to detect differences in EV cargo.

## 7 Remaining Questions in EV Biology

Beyond methodological considerations and rational selection of cell‐type markers, several conundrums remain related to EV biology that have the potential to hinder the clinical translation of tissue‐specific EVs. Firstly, the mechanisms by which EVs cross from the interstitial space into the bloodstream are not fully understood (Iannotta et al. 2024). The types of endothelium present across different organs as well as within the same organ affect the nature and origins of EVs detectable in circulation, as does injury, inflammation, cancer and other conditions that compromise endothelial barriers (Amruta et al. 2023). Although discontinuous endothelium theoretically permits the paracellular passage of EVs in small size ranges (up to 200 nm), various other modes of transport that modify tight junctions or exploit transcellular pathways must be used to traverse continuous or fenestrated endothelium (Iannotta et al. 2024). Although the array of glycoproteins, integrins and other molecules on EV surfaces may facilitate movement through the extracellular matrix and across the endothelium, the passive diffusion of relatively large vesicle particles into the bloodstream is unlikely amid the interstitial fluid gradient, which directs away from capillaries and into lymphatic vessels (Howard et al. 2022; Arif and Moulin 2023). Thus, EVs may alternatively enter the bloodstream at lymphovenous junctions (Iannotta et al. 2024).

The impact of these interactions and transport mechanisms on the EV finally found in the blood is also unknown, raising the question of whether cell‐type‐specific markers are removed or acquired in the process. Indeed, the capacity for EV recipient cells to uptake and re‐release molecular cargo in EVs adds to the challenge of establishing cellular origin (Obata et al. 2018). Adipocytes and endothelial cells were observed to exchange membrane proteins via EVs, replenishing the expression of caveolin‐1 (CAV1) in CAV1‐knockout adipocytes, in a phenomenon termed ‘plasma membrane sharing’ (Crewe et al. 2018). Moreover, emerging observations of multilamellar and multicompartmental vesicular structures in biofluids complicate the specific isolation of EV subpopulations based on surface protein exposure, depending on their arrangement in the membrane layers and how the structure was produced (Broad et al. 2023; Saadeldin et al. 2023). Lastly, as EVs from the same cell type can carry diverse sets of molecular contents, it is plausible that specific cell type markers will be absent from EV subsets or influenced by changes in EV biogenesis and secretion under certain conditions. Continued efforts to unravel EV heterogeneity will be imperative to understanding how representative the enriched EV populations are to their origin and for designing methods to capture this diversity (Carney et al. 2025).

## 8 Limitations of IAC

Although selecting for a subset of EVs based on surface marker expression is useful for highly specific applications, the bias this inherently introduces is also a major limitation of the approach. As surface protein expression on EVs is often heterogeneous, a significant proportion of EVs released by the origin of interest may lack the target marker altogether (Carney et al. 2025), potentially excluding biologically relevant vesicles from the analysis. Increasing the specificity in this way risks the loss of important diagnostic or functional subpopulations and may narrow the representation of the full cellular output of EVs from a tissue. Additionally, isolating tissue‐specific EVs, though valuable for understanding local processes, may fail to capture broader physiological or pathological contexts. Unlike bulk EV analysis, which reflects composite signals from multiple tissues, highly specific enrichment may exclude circulating EVs involved in inter‐organ communication or systemic responses such as inflammation and metabolic regulation (Hu et al. 2023). Accordingly, an IAC‐based approach should be carefully designed to balance tissue specificity with biological relevance, particularly to address a research question for which both localised and systemic insights are diagnostically or mechanistically important.

## 9 Conclusions and Outlook

With continued evolution of EV enrichment strategies, analytical technologies and understanding of the roles of specific EV subpopulations, research in this field promises to deconvolute complex disease mechanisms and reveal new candidates for biomarker development and therapeutics. Enhancing the specificity of liquid biopsies by selectively tracing EVs of relevant origins brings the unique capability to not only limit noise in analyses of biomolecular cargo, but to provide an ongoing window into organ function without invasive tissue biopsies. A variety of biological and technical factors related to the successful IAC of tissue‐specific EVs should be considered in interpreting prior evidence, especially as studies differ in methods for pre‐enriching EVs from biofluids and the degree of characterisation. The suitability of cell‐type markers proposed for detection and isolation of EVs from different origins will depend on the goal of the assay, the need for complete isolation from other cellular sources or tolerance for impurities that negligibly affect readout. Further study is anticipated to define these thresholds and harmonise methods to support the development of robust clinically relevant technologies in the future.

## Author Contributions

**Lauren A Newman**: conceptualization; investigation; writing – original draft; writing – review and editing. **Andrew Rowland**: conceptualization; writing – review and editing.

## Conflicts of Interest

Andrew Rowland and Lauren Newman are recipients of research funding from Pfizer Inc., AstraZeneca and Boehringer Ingelheim for work outside of the scope of this project. The other authors declare no conflicts of interest.

## Data Availability

Data sharing is not applicable to this article as no datasets were generated or analysed during the current study.
